# Emerging Oncogenic and Immunoregulatory Roles of BST2 in Human Cancers

**DOI:** 10.3390/biomedicines14010131

**Published:** 2026-01-08

**Authors:** Chohee Kim, Seoyoon Choi, Jong-Whi Park

**Affiliations:** 1 Department of Health Sciences and Technology, GAIHST, Gachon University, Incheon 21999, Republic of Korea; kims4kim@gachon.ac.kr; 2 Department of BioNano Technology, College of BioNano Technology, Gachon University, Seongnam 13120, Republic of Korea; drdog0508@gachon.ac.kr; 3 Department of Life Sciences, College of BioNano Technology, Gachon University, Seongnam 13120, Republic of Korea

**Keywords:** BST2 (CD317, tetherin), cancer immune regulation, exosome, tumor microenvironment, interferon signaling, hypomethylation, glioblastoma, monoclonal antibody therapy, CAR-T immunotherapy

## Abstract

BST2 has emerged as a multifunctional molecule that bridges antiviral defense, membrane architecture, and tumor immunity. Originally characterized as an interferon-inducible restriction factor that tethers virions to the plasma membrane, BST2 is now recognized as an oncogenic driver and immunoregulatory hub in diverse malignancies. In cancer, BST2 expression is frequently upregulated through promoter hypomethylation and transcriptional activation. Functionally, BST2 promotes proliferation, epithelial–mesenchymal transition, anoikis resistance, and chemoresistance, whereas its loss sensitizes tumor cells to proteotoxic and metabolic stresses. Beyond tumor cells, BST2 modulates the tumor microenvironment by promoting M2 macrophage infiltration, dendritic cell exhaustion, and natural killer (NK)-cell resistance, thereby contributing to immune evasion. Elevated BST2 expression correlates with poor prognosis in glioblastoma, breast, nasopharyngeal, and pancreatic cancers, and it serves as a circulating biomarker within small extracellular vesicles. In conclusion, BST2 is a dual-function molecule that integrates oncogenic signaling and immune regulation, making it an attractive diagnostic and therapeutic target for hematological and solid tumors.

## 1. Introduction

Bone marrow stromal antigen 2 (BST2), also known as CD317, tetherin, or HM1.24, is a type II transmembrane glycoprotein that is broadly expressed in human tissues, including hepatocytes, endothelial cells, pneumocytes, monocytes, and plasma cells. Its expression is modulated by interferons in a cell type- and tissue context- dependent manner [1]. Originally identified as a viral restriction factor that physically tethers budding virions to the plasma membrane, BST2 has now emerged as a multifunctional regulator that integrates antiviral defense, membrane organization, and immune signaling [2,3].

BST2 plays oncogenic and immunoregulatory roles in a wide range of malignancies (Table 1). BST2 expression is elevated in gastric [4,5,6], oral cavity squamous cell [7,8], breast [9,10,11,12,13], cervical [14], pancreatic [15], nasopharyngeal [16,17], head and neck [18], endometrial [19], lung [20], bladder [21], hepatocellular [22], colorectal [23], and thyroid cancers [24], as well as in glioblastoma [25,26,27] and BST2^+^ macrophages in pancreatic tumor microenvironments [28]. Thus, its protumorigenic and immunomodulatory functions are broadly conserved across human cancers.

Overall, BST2 has been redefined as a molecule that transcends its original antiviral role. By linking endomembrane homeostasis, vesicle trafficking, and immune regulation, BST2 functions as a pleiotropic modulator that contributes to tumor cell survival as well as immune evasion. The present review summarizes the emerging aspects of BST2 biology, emphasizing its structural features, regulatory mechanisms, and oncogenic–immunoregulatory functions in hematologic and solid malignancies.

## 2. Structural and Molecular Features of BST2

**Figure 1:**
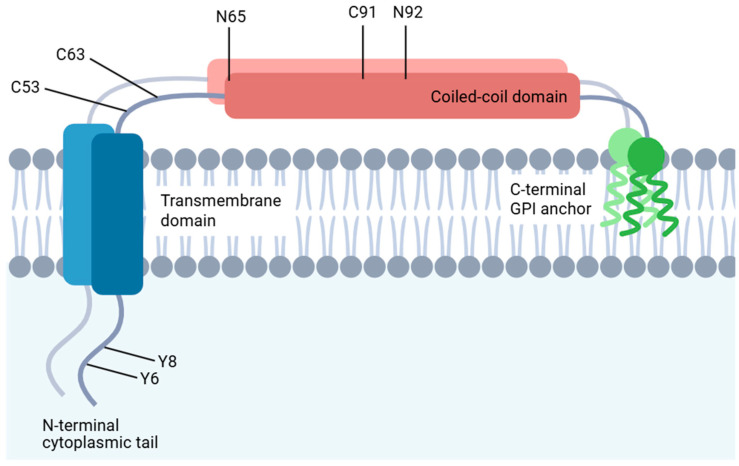
Structural topology of BST2 (CD317 or tetherin). Created in BioRender. Park, J. (2026). http://BioRender.com/r2a494t. BST2 is a type II membrane protein characterized by an N-terminal transmembrane domain, a coiled-coil ectodomain, and a C-terminal GPI anchor. Key structural motifs include cysteine residues (C53, C63, and C91), which are essential for dimerization, N-linked glycosylation sites (N65 and N92), and a dual-tyrosine motif (Y6 and Y8) that mediates endocytosis and signaling.

BST2 is a membrane protein with an unusual topology that is simultaneously anchored via an N-terminal transmembrane domain and a C-terminal glycosylphosphatidylinositol (GPI) anchor [29] (Figure 1). This unique dual-anchoring configuration underlies a functional bifurcation in which the GPI anchor is essential for restricting viral release, whereas NF-κB signaling depends on distinct structural elements, such as the cytoplasmic YxY motif [30]. Beyond its static structural roles, functional assays have revealed that BST2 traffics dynamically through the trans-Golgi network, indicating regulated membrane recycling [29,31]. Unlike most GPI-anchored proteins, BST2 undergoes clathrin-mediated endocytosis from lipid rafts. This internalization is governed by a non-classical dual tyrosine motif (Tyr-6 and Tyr-8) located within its N-terminal cytoplasmic tail [31]. Intriguingly, substituting the cytoplasmic tyrosine residues Y6 and Y8 of BST2 with alanine disrupts its pro-migratory and pro-invasive functions in breast cancer cells [32]. The N-terminal cytosolic region of BST2 also functions as a microdomain exclusion signal, thereby reducing raft association when appended to heterologous proteins. Although the long and short isoforms of BST2 exhibit equivalent microdomain affinities, they differ markedly in their ability to activate NF-κB signaling, indicating functional divergence [33].

The cysteine-linked dimerization of BST2, mediated by residues C53, C63, and C91, is essential for inhibiting HIV-1 release, whereas N-linked glycosylation at N65 and N92 is dispensable for its antiviral function [34]. Dimeric BST2 enhances breast cancer cell adhesion and survival in suspension through GRB2/extracellular signal-regulated kinase (ERK)-mediated phosphorylation and proteasomal degradation of the proapoptotic protein BIM. Conversely, the disruption of BST2 dimerization compromises the tumorigenic capacity by impairing anoikis resistance, anchorage-independent growth, and in vivo metastatic potential [35].

Biophysical analyses have demonstrated that the BST2 ectodomain exhibits considerable conformational flexibility, which may be essential for accommodating the membrane curvature observed during viral budding [36]. Loss of BST2 expression results in the disorganization of lipid microdomains and reduced membrane order, supporting its proposed role in forming a ‘tethered picket fence’ that stabilizes the membrane architecture. In this model, BST2 functions as a structural anchor that links membrane rafts to the actin cytoskeleton, thereby restricting lateral diffusion and organizing membrane domains [37]. Consistent with this structural tethering function, BST2 is required to anchor midbody remnants (MBRs) to the cell surface following cytokinesis. BST2 depletion leads to an increased release and intercellular transfer of MBRs, underscoring its pivotal role in promoting local retention and limiting the extracellular dissemination of these organelles [38]. A recent study has revealed that BST2 directs calnexin to RACK1-mediated autophagic degradation, thereby preserving calcium balance and an optimal protein-folding environment in the ER, while mitigating proteotoxic stress and apoptosis induced by proteasome inhibitors [39].

## 3. Regulation of BST2 Expression

**Figure 2:**
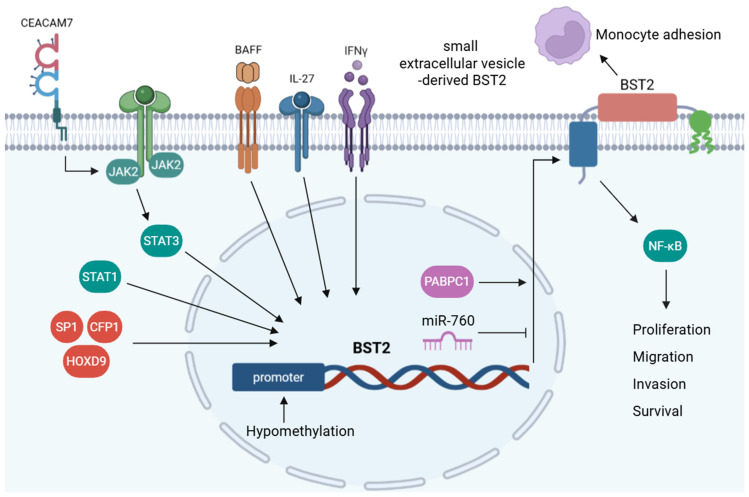
Regulation and functions of BST2. Created in BioRender. Park, J. (2026) http://BioRender.com/3z9k00l. BST2 expression is modulated by cytokines (IFN-γ, IL-27, and BAFF), transcriptional factors (SP1, HOXD9, and STAT1/3), and promoter hypomethylation. Meanwhile, PABPC1 and miR-760 regulate its post-transcriptional control. BST2 mediates monocyte adhesion and activates NF-κB signaling to promote tumor cell proliferation, migration, and survival. The extracellular form of BST2, including sEV-associated BST2, functions as a secreted biomarker that further enhances tumor progression through intercellular communication within the tumor microenvironment.

BST2 expression is tightly regulated by immune cues and epigenetic mechanisms (Figure 2). Various cytokines modulate BST2 levels across distinct cell types, underscoring the molecule’s broad involvement in immune and inflammatory processes [40,41,42]. Interferon gamma (IFN-γ) selectively induces robust BST2 expression in human endothelial cells, where membrane-bound BST2 mediates monocyte adhesion, whereas the purified BST2 extracellular domain significantly suppresses this adhesion by competitive inhibition [40]. Interleukin (IL)-27 upregulates BST2 in sensory neurons and keratinocytes and promotes neuronal BST2 release. Recombinant BST2 elicits acute pruritic behavior following intradermal injection [41]. In neoplastic B-lymphoid cells, BST2 expression is upregulated by B-cell activating factor (BAFF), promoting NF-κB-dependent survival and proliferation. BST2 silencing attenuates BAFF-mediated NF-κB activation, whereas pharmacological inhibition of NF-κB recapitulates the anti-proliferative and proapoptotic effects of BST2 knockdown [42].

Genome-wide integrative methylation and expression analyses have revealed that BST2 is epigenetically upregulated in glioblastomas because of promoter hypomethylation [25]. BST2 DNA hypomethylation at specific CpG sites correlates inversely with its elevated expression in breast cancer, and treatment with 5-azacytidine further increases BST2 expression in low-expressing breast cancer cells but has no effect on cells with already high expression [10]. Similarly, BST2 is upregulated in cervical cancer owing to promoter hypomethylation and direct transcriptional activation by STAT1. Functionally, BST2 enhances tumor progression by promoting proliferation, epithelial–mesenchymal transition (EMT), and apoptosis resistance, whereas its suppression via STAT1 silencing or methylation restoration attenuates these malignant phenotypes [14].

BST2 expression is tightly regulated by various transcription factors that drive cancer progression and metastatic potential [5,15,16,43]. SP1 functions as a transcriptional activator of BST2, and its overexpression increases BST2 levels and promotes oncogenic behavior. Conversely, silencing BST2 reverses the SP1-driven proliferative and migratory effects, highlighting the SP1–BST2 axis as a potential therapeutic target in pancreatic cancer [15]. CFP1 directly binds to the BST2 promoter and modulates H3K4me3 deposition, which is essential for the maintenance of ovarian cancer cell growth [43]. HOXD9 and PABPC1 promote gastric cancer metastasis through transcriptional activation and post-transcriptional stabilization of BST2, respectively. BST2 silencing inhibits HOXD9- and PABPC1-driven EMT and invasiveness in gastric cancer cells [5]. Carcinoembryonic antigen-related cell adhesion molecule 7 (CEACAM7)-induced activation of the JAK2/STAT3 signaling cascade leads to transcriptional upregulation of BST2, which is crucial for promoting nasopharyngeal carcinoma (NPC) cell motility. Pharmacological inhibition of JAK2/STAT3 or silencing of BST2 suppresses CEACAM7-driven migration in NPC cells [16].

Loss of TGFβ-AP2-mediated repression in high-grade breast cancer leads to persistent BST2 expression, thereby conferring apoptosis resistance and increased cell proliferation. Consistent with this, the pharmacological suppression of BST2 by resveratrol or genetic knockdown restores apoptotic sensitivity and inhibits tumor cell proliferation, as resveratrol reinstates AP2-mediated transcriptional repression of BST2 [11]. Intriguingly, acetyl-11-keto-β-boswellic acid (AKBA) was identified as a functional BST2-binding molecule that mediates protective effects in human pulmonary microvascular endothelial cells. AKBA upregulates BST2 transcription by activating unphosphorylated STAT1 [44]. In addition to transcriptional regulation, miRNA-mediated mechanisms also modulate BST2 expression. Regulatory axis between miR-760 and BST2 modulates matrix metalloproteinase activity and apoptotic signaling. Although targeting BST2 with miR-760 enhances apoptosis and reduces tumor growth, miR-760 exerting additional effects via BST2-independent pathways is plausible [45].

## 4. Antiviral Functions of BST2

BST2 acts as an IFN-α-induced host restriction factor that physically prevents the release of retroviral particles by anchoring them onto the host cell membrane. The antiviral activity of BST2 depends on specific structural features, particularly cysteine-linked dimerization, and the integrity of its GPI anchor and cytoplasmic tail, which are essential for restricting retroviral particle release [34,46]. Human BST2 senses retained HIV-1 particles at the plasma membrane and triggers a robust NF-κB-dependent proinflammatory response through surface clustering and the subsequent recruitment of TRAF6 and TAK1 [47]. Perez-Caballero et al. have demonstrated that the structural configuration, rather than a specific amino acid sequence, is critical for BST2’s antiviral function by constructing a synthetic BST2-like protein. Moreover, the HIV-1 accessory protein, Vpu, counteracts BST2 by preventing its incorporation into the viral membrane, thereby abolishing its tethering activity [48]. LC3C, a specific ortholog of the ATG8 family, interacts selectively with the HIV-1 accessory protein Vpu and is essential for the Vpu-mediated antagonism of BST2. This process proceeds via a non-canonical autophagy pathway that requires ATG5 and Beclin-1 [49]. ATG5 directly binds to virus-tethered, phosphorylated (Tyr-6 and Tyr-8), and dimerized BST2 at the plasma membrane, initiating its internalization and degradation. This ATG5–BST2 interaction occurs independently of HIV-1 Vpu and LC3C and does not require ATG5’s conjugation with ATG12, revealing an autophagy-independent role for ATG5 in viral immune evasion [50].

In neurons, BST2 is predominantly localized in non-synaptic compartments. Unlike its well-established antiviral function in non-neuronal cells, neuronal BST2 facilitates measles virus replication, leading to increased viral accumulation in murine brain tissue and primary neuronal cultures. BST2-deficient mice and neurons exhibit significantly reduced levels of measles virus RNA and protein [51]. BST-2 restricts hepatitis B virus (HBV) by tethering virions within multivesicular bodies, whereas HBV counteracts this restriction via HBx-induced intracellular accumulation of BST2 in hepatocytes, a mechanism distinct from that of HIV-1 Vpu [52]. In influenza A-infected cells, BST2 promotes IRE1α-dependent splicing of XBP-1, leading to cytochrome c release and caspase activation, thereby inducing apoptosis. Conversely, BST2-deficient cells exhibit reduced levels of apoptotic markers and increased viral particle production, indicating that BST2 suppresses viral replication through cell death induction rather than viral tethering alone [53].

Similarly, BST2 overexpression markedly inhibits cell–cell fusion and replication of measles virus by decreasing the levels of the hemagglutinin glycoprotein without affecting viral entry or receptor expression [54]. In addition, porcine epidemic diarrhea virus (PEDV) infection induces the expression of BST2 through direct transcriptional activation by interferon regulatory factor 1, independent of interferon signaling. Subsequently, BST2 restricts PEDV replication by targeting the viral nucleocapsid (N) protein for selective autophagic degradation via MARCHF8-mediated ubiquitination [55]. Collectively, these findings highlight BST2 as a versatile antiviral effector that employs diverse virus-specific mechanisms, ranging from virion tethering and autophagic degradation to apoptosis induction, to restrict viral replication and dissemination.

**Figure 3:**
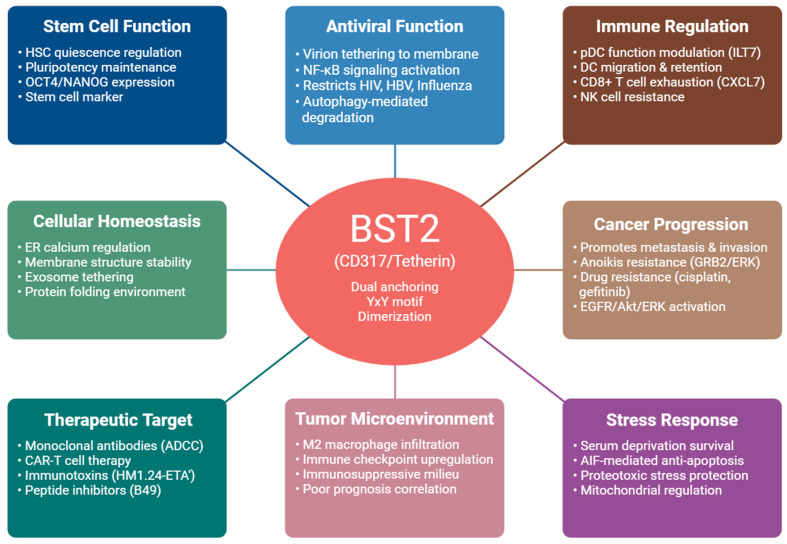
Structure–trafficking–signaling–phenotype framework of BST2. BST2 structural features, including dual membrane anchoring, dimerization, and YxY motif-dependent trafficking, enable membrane microdomain organization and engagement of shared signaling pathways. These properties underlie BST2’s context-dependent antiviral, oncogenic, and immunoregulatory functions.

Importantly, the structural features that underlie BST2’s antiviral activity—dual membrane anchoring, cysteine-linked dimerization, and YxY motif-dependent trafficking—also provide a mechanistic basis for its oncogenic and immunoregulatory functions. These conserved membrane-organizing properties are repurposed in cancer cells to modulate receptor compartmentalization, activate ERK and AKT/mTOR signaling, and enhance resistance to cellular stress. This structure–trafficking–signaling framework linking BST2’s antiviral and cancer-related roles is summarized in Figure 3.

## 5. BST2 in Cancer Biology

### 5.1. Hematological Malignancies

The humanized anti-BST2 monoclonal antibody elicits potent antibody-dependent cellular cytotoxicity (ADCC) against myeloma cells, both in established cell lines and patient-derived tumor samples, when cocultured with peripheral blood mononuclear cells, highlighting its therapeutic potential in multiple myeloma [56]. A novel immunotoxin, HM1.24-ETA’, comprising a BST2-specific single-chain variable fragment fused to a truncated *Pseudomonas* exotoxin, effectively induces apoptosis in BST2-positive myeloma cells in vitro and significantly prolongs survival in a severe combined immunodeficiency (SCID) mouse model [57].

### 5.2. Antibody-Based Immunotherapy in Lung Cancers

Building on these findings in hematological cancers, subsequent studies have identified BST2 as a key player in multiple solid tumors with diverse tumor-promoting functions. Chimeric and humanized anti-BST2 monoclonal antibodies triggered robust ADCC against BST2-positive lung cancer cells, with NK cells serving as primary effectors. A direct correlation was observed between BST2 surface expression levels and the efficacy of ADCC, and pretreatment with cytokines, such as IL-15 and IFN-γ, significantly enhanced cytotoxic effects [58]. In SCID mouse models, anti-BST2 antibody therapy significantly reduced tumor growth, highlighting its potential clinical application in lung cancer immunotherapy [20] (Table 2).

### 5.3. BST2 in Breast and Metastatic Progression

BST2 expression is significantly upregulated in mouse mammary tumor virus-induced mammary tumors independent of canonical interferon signaling [62]. The co-expression of sialyl-Lewis x, a carbohydrate epitope that mediates tumor–endothelium adhesion via E-selectin binding, and BST-2 defines a subset of patients with estrogen receptor-negative breast cancer at an increased risk for aggressive metastasis, particularly to the liver and brain [63]. In addition, BST2 expression is markedly elevated in bone metastatic breast cancer cell lines and tissue specimens compared to that in their non-metastatic counterparts, and serum BST2 levels exhibit high sensitivity and specificity as a potential biomarker for detecting bone metastasis in patients with breast cancer [12]. BST2 overexpression not only promotes invasive and migratory behavior in tamoxifen-resistant breast cancer cells in vitro, but also significantly enhances metastatic nodule formation in the lungs when overexpressed in melanoma cells in vivo, indicating its pivotal role in both cellular aggressiveness and systemic metastatic progression [13]. Using basement membrane matrix embedment and competitive experimental pulmonary metastasis assays, BST2 suppression markedly impairs invadopodia formation, extracellular matrix degradation, and the efficiency of metastatic colonization in the lungs of mouse models [64].

### 5.4. BST2 in Glioma and Brain Tumors

BST2 is upregulated in glioblastoma and negatively correlated with tumor purity [25,26]. Wainwright et al. have demonstrated that BST2 is predominantly expressed in tumor cells but not in infiltrating immune cells within brain tumors. Despite its elevated expression in gliomas, neither genetic knockdown nor antibody-based targeting of BST2 alters survival outcomes in an orthotopic syngeneic GL261 mouse model [65]. Subsequent studies using human BST2 validated it as a stable and glioma-specific surface antigen, enabling the development of BST2-directed CAR-T cells with potent antitumor activity in vitro and in orthotopic human glioma xenograft models. To prevent BST2-mediated fratricide in CAR-T cells, shRNA-based silencing of BST2 has been employed to enhance cell viability, persistence, and long-term effector function [60]. This discrepancy may reflect fundamental differences between mouse and human glioma models, as well as modality-specific mechanisms distinguishing antibody-based targeting from CAR-T–mediated cytotoxicity. More recently, BST2 and DIRAS3 were identified as critical mediators of cytotoxic T cell immune evasion and were independently correlated with adverse prognosis in high-grade gliomas. Functional silencing of BST2 and DIRAS3 significantly impairs glioma cell invasion and migration, supporting their role in malignant progression [27].

### 5.5. BST2 in Other Solid Tumors

BST2 serves as a secreted biomarker in several solid tumors. Enzyme-linked immunosorbent assay and immunohistochemistry verified the significant elevation of tumor-secreted BST2 in both the plasma and tissues of patients with colorectal cancer, identifying BST2 as a promising non-invasive diagnostic marker for early detection and disease monitoring [23]. Similarly, a serum proteomics approach has revealed BST2 enrichment in small extracellular vesicles (sEVs) from patients with papillary thyroid microcarcinoma associated with lymph node metastasis. Functional assays confirmed that sEV-derived BST2 promotes cancer cell proliferation, migration, and lymphatic endothelial tube formation in vitro and metastasis in vivo [24]. In addition, BST2 anchors exosomes to the plasma membrane through its C-terminal GPI anchor, and its depletion markedly reduces surface-bound exosomes while enhancing their extracellular release [66].

BST2 also exerts oncogenic signaling effects in multiple solid tumors. In bladder cancer, BST2 expression is markedly elevated, particularly in the muscle-invasive subtypes. Its suppression impairs proliferation and attenuates Akt and ERK phosphorylation, whereas enforced BST2 expression enhances proliferative capacity and activates downstream signaling pathways [21]. In hepatocellular carcinoma, BST2 facilitates the release of EGFR from lipid rafts, enabling ligand-independent receptor activation and promoting tumorigenicity [22]. Consistent with this mechanism, BST2 upregulation in oral squamous cell carcinoma contributes to gefitinib resistance by activating the EGFR–Akt/ERK signaling axis [8].

BST2 also promotes the survival of cancer cells under metabolic stress. Under serum deprivation conditions, BST2 inhibits apoptosis through a mitochondria-dependent AIF-mediated pathway that operates independently of caspases and autophagy. Conversely, BST2 silencing causes mitochondrial membrane depolarization and AIF nuclear translocation, thereby enhancing apoptosis, specifically under nutrient-deprived conditions [67]. In NPC, BST2 confers cisplatin resistance by activating NF-κB signaling and upregulating anti-apoptotic genes, such as Bcl-XL and Livin. Clinically, high BST2 expression levels correlate with poor prognosis in patients with NPC treated with platinum-based chemotherapy [17].

## 6. BST2 and Immune Regulation

### 6.1. Immune Cell Regulation

While transcriptional regulators such as IRF4 govern the intrinsic differentiation and persistence of antitumor CD8^+^ T cells [68], BST2 shapes the extrinsic tumor and immune microenvironment that ultimately determines the effectiveness of T cell–based immunotherapies. The type I IFN-inducible protein BST2 has been identified as a direct ligand for ILT7, and its interaction leads to the potent downregulation of TLR7/9-mediated cytokine production by plasmacytoid dendritic cells (pDCs). The ILT7–BST2 axis represents a regulatory feedback mechanism in antiviral immunity and tumor environments, with potential relevance in modulating pDC function in autoimmune diseases and cancer [69]. In line with this, BST2 antibody-mediated depletion of pDCs in a transgenic HNSCC model significantly impeded tumor growth and revitalized antitumor immunity by expanding effector T cells and reducing regulatory T cells and monocytic myeloid-derived suppressor cells. [70]. Combined treatment with CpG oligodeoxynucleotides and anti-BST2 antibodies further enhances antitumor efficacy through immune-mediated synergy, characterized by increased infiltration of NK cells and macrophages into the tumor microenvironment, as shown by FACS analysis in SCID mice [61].

In tumor tissues, an elevated expression pattern is associated with an immunosuppressive microenvironment characterized by elevated immune checkpoint activity and increased M2 macrophage infiltration, which collectively contribute to a poor patient prognosis [26]. Yang et al. have demonstrated that BST2 acts as a key immunoregulatory molecule in HNSCC, showing significant co-expression with immune checkpoint molecules (PD-L1, B7-H3, and B7-H4) and tumor-associated macrophage markers (CD68 and CD163) [18]. Similarly, in endometrial cancer, BST2 has been validated as a potential immunotherapeutic target, with anti-BST2 monoclonal antibodies eliciting strong antitumor responses via ADCC and complement-dependent cytotoxicity (CDC) [19].

In the pancreatic tumor microenvironment, BST2^+^ macrophages enriched via IFN-α signaling correlate with poor prognosis and drive CD8^+^ T cell exhaustion. Mechanistically, BST2 activates ERK signaling to induce CXCL7, which binds to CXCR2 and promotes CD8^+^ T cell dysfunction through the AKT/mTOR pathway [28]. In addition, BST2 enhances tumor cell resistance to NK cell- and CAR-NK-mediated cytotoxicity by maintaining plasma membrane integrity. This protective mechanism functions independent of immune effector activation and requires cytoskeletal organization through BST2’s interaction with RICH2, underscoring its role in immune evasion [71].

During early retroviral infection, BST2 enhances dendritic cell immunogenicity, promoting the upregulation of MHC-II and CD80 in infected mice. Although BST2 does not directly inhibit acute viral replication, it facilitates the early activation of NK and CD4^+^ T cells through improved antigen presentation and IL-15 induction [72]. Furthermore, BST2 promotes effective immune activation by enhancing the migration and retention of conventional dendritic cells within regional lymph nodes via the regulation of CCR7 and ICAM-1 expression. In its absence, antigen-specific adaptive immune responses are weakened, leading to reduced vaccine effectiveness against viral infections and diminished disease severity in autoimmune arthritis models [73]. In addition, BST2 enhances the immunoregulatory function of mesenchymal stem/stromal cells (MSCs) by stabilizing the TNFR1 complex within lipid rafts. This promotes heightened TNF-α responsiveness and activation of the NF-κB–TSG6 signaling axis. Conversely, BST2 loss disrupts this cascade, leading to diminished TSG6 expression and compromised therapeutic efficacy of MSCs in inflammatory disease models [74].

### 6.2. BST2-Driven Quiescence—Pluripotency Switch

IFN-γ disrupts hematopoietic stem cells (HSCs) quiescence by inducing BST2 expression, which displaces HSCs from their quiescent, CXCL12-rich niches in the bone marrow. BST2 enhances E-selectin binding and facilitates HSC relocalization, while also being required for IFN-γ-induced HSC activation through ERK1/2 phosphorylation and lipid raft polarization. In the absence of BST2, HSCs exhibit impaired IFN-γ responsiveness, resist proliferative activation, and retain quiescence, accompanied by altered transcriptional and proteomic profiles within the MAPK/ERK signaling axis [75,76]. Beyond hematopoietic regulation, BST2 functions as a conformation-sensitive surface marker that is widely present in both naïve and primed human pluripotent stem cells (hPSCs). BST2 knockdown in hPSCs leads to a significant reduction in pluripotency factors, such as OCT4 and NANOG, underscoring its necessity for maintaining an undifferentiated state [77]. Moreover, BST2 has been identified as a novel, specific surface marker capable of reliably isolating conjunctival epithelial stem/progenitor cells from the human-induced pluripotent stem cell-derived ocular surface epithelium. BST2^+^ cells exhibit high clonogenic potential and differentiate efficiently into epithelial sheets containing both conjunctival epithelial and goblet cells, confirming their regenerative capacity [78].

## 7. Therapeutic Approaches Targeting BST2

BST2 monoclonal antibodies have demonstrated potent antitumor efficacy against multiple malignancies, initially in multiple myeloma and later in lung and endometrial cancers, by triggering immune-mediated cytotoxicity (ADCC and CDC). When combined with immunostimulatory agents, such as cytokines or CpG oligodeoxynucleotides, these antibodies further enhance NK cell- and macrophage-dependent tumor clearance, highlighting BST2 as a promising immunotherapeutic target [19,56,58,61]. In both IFN-α-sensitive and -insensitive renal cell carcinoma xenograft models, co-administration of IFN-α with either humanized or murine anti-BST2 antibodies significantly enhances antitumor efficacy [79].

BST2-targeted therapeutics have expanded beyond monoclonal antibodies to include HM1.24-ETA’ immunotoxins that selectively induce apoptosis in BST2^+^ tumor cells, as well as BST2-directed CAR-T cells engineered with shRNA to prevent fratricide, both demonstrating potent and specific antitumor activity in preclinical models [57,60]. Peptide B49 and its analog B49Mod1 specifically disrupt BST2-mediated adhesion, effectively impairing cell–cell and cell–matrix interactions. Furthermore, B49 treatment significantly reduces tumor growth and improves survival in a syngeneic mouse model without detectable toxicity [59].

## 8. Conclusions

BST2 has evolved from an interferon-induced viral restriction factor into a multifunctional molecule that integrates oncogenic signaling, membrane dynamics, and immune regulation. In addition to its established antiviral and immunomodulatory functions, BST2 maintains intracellular homeostasis and intercellular communication, which are essential for tumor adaptation and survival. These findings underscore BST2 as a critical coordinator of proteostasis, vesicular trafficking, and tumor microenvironment signaling. Collectively, the dual role of BST2 in intracellular stress adaptation and extracellular communication establishes it as an attractive therapeutic target and prognostic biomarker for diverse malignancies.

## Figures and Tables

**Table 1:** Expression patterns of BST2 across human cancers.

Cancer Type	Sample Type	Methods	Expression Pattern	Comparison Group	[Refs.]
Gastric	GC, CRC, ESCC tissues	IHC	Increased	Non-neoplastic mucosa	[4]
	GC tissues and cell lines (MKN-74, MGC-803, MKN-45, AGS, NCI-N87, HGC-27, SNU-719)	WB, IHC	Increased	Normal gastric epithelium and cell line (GES-1)	[5]
	GC tissues and cell lines (NCI-N87, MGC-803, SGC-7901, BGC-823)	WB, IHC	Increased	Normal tissues and gastric epithelium cell line (GES-1)	[6]
Oral cavity squamous cell	OSCC tissues	qRT-PCR, IHC, WB	Increased	Adjacent normal tissues	[7,8]
Breast	Breast-invasive carcinoma data (TCGA)	Transcriptomic analysis	Increased	Normal breast tissues	[9]
	Breast cancer tissues (TCGA)	Transcriptomic analysis	Increased	Normal mammary gland tissues	[10]
	Primary breast cancer cell lines	qRT-PCR	Increased	Non-malignant primary breast epithelial cells	[11]
	Primary human breast cancer cell lines (MDA-231, HTB-121, BC-701, UACC812, MCF-7, T47D, MDA-468), Bone metastatic breast cancer cell line (MDA-231BO)	WB, qRT-PCR, ELISA, cDNA microarray analysis	Increased	Normal breast cell line (MCF-10A)	[12]
	Tamoxifen-resistant MCF-7 human breast cancer cell line (TRM-7)	WB, qRT-PCR	Increased	Human breast cancer cell line (MCF-7)	[13]
Cervical	Cervical cancer tissues and cell lines (HeLa cells, SiHa cells)	Transcriptomic analysis, WB, qRT-PCR	Increased	Normal tissue and cervical epithelial cells (HcerEpic)	[14]
Pancreatic	Pancreatic cancer cell lines (SW1990, BxPC3, PANC1, PSN-1)	Transcriptomic analysis, WB, qRT-PCR	Increased	Human pancreatic duct epithelial cell line (HPDE6-C7)	[15]
Nasopharyngeal	Nasopharyngeal carcinoma (NPC) tissues (GSE12452, GSE53819)	Transcriptomic analysis	Increased	Normal tissues	[16]
	Cisplatin-resistant NPC cell lines (CNE2)	WB	Increased	Cisplatin-sensitive NPC cell lines (S16)	[17]
Head and neck	Primary head and neck squamous cell carcinoma (HNSCC) tissues	IHC	Increased	Adjacent normal mucosa	[18]
Endometrial	Endometrial cancer cell lines (HEC-1, HEC-1A, HEC-6, HEC-88nu, HEC-108, HEC-116, HEC-251, SNG-II, SNG-M)	qRT-PCR, Flow cytometry, IHC	Increased	Normal endometrial cell line (EM-E6/E7/TERT)	[19]
Lung	Human lung cancer cells	Flow cytometry	unchanged	Multiple myeloma cells (RPMI8226)	[20]
Bladder	8 urothelial carcinoma (UC) tissues	qRT-PCR, IHC	Increased	Normal lung, stomach, liver, bone marrow tissues	[21]
Hepatocellular	Hepatocellular carcinoma tissues, Human protein atlas	Proteomic analysis, IHC	Increased	Normal liver tissues	[22]
Colorectal	CRC tissues, plasma samples	IHC, ELISA	Increased	Adjacent nontumor epithelial cells	[23]
Thyroid	Small extracellular vesicles (sEVs) from papillary thyroid microcarcinoma tissues	WB, ELISA	Increased	sEVs from benign thyroid nodule	[24]
Glioblastoma	GBM tissues	Transcriptomic analysis	Increased	Non-neoplastic brain tissues	[25]
	GBM tissues (TCGA-glioma dataset)	Transcriptomic analysis	Increased	Non-neoplastic brain tissues	[26]
	Glioma cell lines (LN229, U87MG)	qRT-PCR, WB	Increased	Normal human astrocytes (svgP12)	[27]

Abbreviation: WB, Western blot; IHC, Immunohistochemistry

**Table 2:** Experimental evidence supporting BST2 as a therapeutic target in human cancers.

Cancer Type	Model	Experimental Setting	Treatment Response/Main Function	[Refs.]
Hematological malignancies	BST2-positive myeloma cells	in vitro	HM1.24-ETA’ induces apoptosis.	[57]
	SCID mice	in vivo	HM1.24-ETA’ prolongs survival.	[57]
Lung cancers	BST2-positive lung cancer cells	in vitro	Antibody-based immunotherapy triggers robust ADCC and CDC.	[58]
	SCID mice	in vivo	Antibody-based immunotherapy reduces tumor growth.	[20]
Breast cancers	Tamoxifen-resistant breast cancer cells	in vitro	BST2 promotes invasive and migratory behavior.	[13]
	Breast cancer cells	in vitro	BST-2-based peptide (B49) inhibits cell adhesion.	[59]
	Syngeneic breast cancer mouse model	in vivo	B49 reduces the rate of breast tumor growth by 35%.	[59]
Glioma	Orthotopic glioma xenograft models	in vivo	CAR-T cells enhance antitumor activity.	[60]
Endometrial cancers	Endometrial cancer cells	in vitro	Anti-BST2 antibodies elicit ADCC and CDC.	[19]
	SCID mice (but not in NOG mice)	in vivo	CpG ODNs enhance anti-BST2 antibody activity.	[61]
Metastatic progression	Lung metastasis mouse model	in vivo	BST2 enhances metastatic nodule formation in the lungs.	[13]
	Lymph node metastasis (LNM) mouse model	in vivo	BST2 promotes lymph node enlargement and metastasis	[24]

## Data Availability

No new data were created or analyzed in this study.
